# Low-mass molecular dynamics simulation for configurational sampling enhancement: More evidence and theoretical explanation

**DOI:** 10.1016/j.bbrep.2015.08.023

**Published:** 2015-09-02

**Authors:** Yuan-Ping Pang

**Affiliations:** Computer-Aided Molecular Design Laboratory, Mayo Clinic, Stabile 12-26, 200 First Street SW, Rochester, MN 55905, USA

**Keywords:** CαβRMSD, Cα and Cβ root mean square deviation, Δ*t*, Time step, LMT, Low-mass time, MD, Molecular dynamics, NTP, Isothermal–isobaric, SD, Standard deviation, SE, Standard error, SMT, Standard-mass time, *T*, Temperature, Molecular dynamics simulation, Protein folding, Folding rate, Folding time, Chignolin, CLN025

## Abstract

It has been reported recently that classical, isothermal–isobaric molecular dynamics (NTP MD) simulations at a time step of 1.00 fs of the standard-mass time (Δ*t*=1.00 fs^smt^) and a temperature of ≤340 K using uniformly reduced atomic masses by tenfold offers better configurational sampling than standard-mass NTP MD simulations at the same time step. However, it has long been reported that atomic masses can also be increased to improve configurational sampling because higher atomic masses permit the use of a longer time step. It is worth investigating whether standard-mass NTP MD simulations at Δ*t*=2.00 or 3.16 fs^smt^ can offer better or comparable configurational sampling than low-mass NTP MD simulations at Δ*t*=1.00 fs^smt^. This article reports folding simulations of two β-hairpins showing that the configurational sampling efficiency of NTP MD simulations using atomic masses uniformly reduced by tenfold at Δ*t*=1.00 fs^smt^ is statistically equivalent to and better than those using standard masses at Δ*t*=3.16 and 2.00 fs^smt^, respectively. The results confirm that, relative to those using standard masses at routine Δ*t*=2.00 fs^smt^, the low-mass NTP MD simulations at Δ*t*=1.00 fs^smt^ are a simple and generic technique to enhance configurational sampling at temperatures of ≤340 K.

## Highlights

• 160 MD simulations were performed to fold chignolin and CLN025 from scratch.
• Such simulations used the following conditions to determine sampling efficiency.
• Reducing atomic masses by tenfold or increasing time step from 1 to 2 or 3.16 fs.
• Equal sampling in simulations using low masses and using the 3.16-fs time step.
• Better sampling in simulations using low masses than using the 2-fs time step.

## 1 Introduction

It has been reported recently that use of uniformly reduced atomic masses by tenfold (hereafter abbreviated as low masses) can enhance configurational sampling in classical, isothermal–isobaric molecular dynamics (NTP MD) simulations at a time step (Δ*t*) of 1.00 fs of the standard-mass time (fs^smt^) [1]. The reported sampling enhancement was determined by the smaller number of time steps required for the low-mass simulations to capture the folding of β-hairpin CLN025 [2] than that of the standard-mass NTP MD simulations performed at the same time step [1]. The folding of CLN025 in the low-mass simulations [1] was verified by the native-state conformation of CLN025 that was independently determined by an NMR spectroscopic study [2].

It has long been reported that increasing atomic masses can improve configurational sampling because higher atomic masses can reduce the fastest motions of the system in a molecular dynamics (MD) simulation to lengthen the Δ*t* of the simulation and can gain larger momenta [3], [4], [5], [6], [7], [8], [9], [10], [11], [12], [13], [14], [15], [16], [17], [18], [19], [20], [21], [22], [23], [24], [25], [26]. Downscaling the solvent mass can reportedly enhance the configurational sampling of oligopeptides in MD simulations through reduction of solvent viscosity [27], [28]. Differentially downscaling the solvent and side-chain masses also reportedly improved configurational sampling of a nonapeptide in MD simulations through reduction of solvent viscosity and adiabatic decoupling of motions of solvent, backbone, and side-chain [29].

Further, it has been reported that scaling the total mass by a factor of 10 for an MD simulation scales the time of the new system by a factor of $\sqrt{10}$ [15], [30]. In theory, as explained in Section 2.1, low-mass MD simulations at Δ*t*=1.00 fs^smt^ are equivalent to standard-mass MD simulations at Δ*t*=$\sqrt{10}$ fs^smt^. In practice, it is reasonable to expect that low-mass NTP MD simulations at Δ*t*=1.00 fs^smt^ can be numerically equivalent to a standard-mass NTP MD simulations at Δ*t*=3.16 fs^smt^ since MD simulations are typically performed with the relatively high double-precision floating-point format. On one hand, because the SHAKE algorithm for bond constraint tends to fail when using Δ*t*≥2.00 fs^smt^[15], the routine Δ*t* of an MD simulation using AMBER MD programs with the SHAKE algorithm has long been 2.00 fs^smt^ at a temperature (*T*) of ≤300 K (AMBER 14 reference manual, p289). On the other hand, according to the results reported in Ref. [1], standard-mass NTP MD simulations with the SHAKE algorithm could be performed at Δ*t*=3.16 fs^smt^ and *T*≤340 K without the use of the hydrogen mass repartitioning scheme that is developed to avoid system instability caused by the use of Δ*t*≥2.00 fs^smt^[15], [19], [26].

In this context, three aims were set to investigate (*i*) whether standard-mass NTP MD simulations at Δ*t*>2.00 fs^smt^ can actually be performed without the use of the hydrogen mass repartitioning scheme, (*ii*) whether the configuration sampling efficiency of standard-mass NTP MD simulations at Δ*t*=3.16 fs^smt^ is statistically equivalent to that of low-mass NTP MD simulations at Δ*t*=1.00 fs^smt^, and (*iii*) whether low-mass NTP MD simulations at Δ*t*=1.00 fs^smt^ can statistically offer better configurational sampling than standard-mass NTP MD simulations at Δ*t*=2.00 fs^smt^.

This article reports a comparative study of the three aims using 160 unique, independent, all-atom, and classical NTP MD simulations—each of which was performed with the SHAKE algorithm for 500×10^6^ time steps—to determine relative configurational sampling efficiencies of using mass scaling factors of 1.0 and 0.1 and time steps of 1.00, 2.00, 3.16, and 3.50 fs^smt^. CLN025 and chignolin [31] were used as model systems for NTP MD simulations of miniprotein folding. To investigate the configurational sampling efficiency in a forcefield independent manner, two forcefields were used in this study—an up-to-date general-purpose AMBER forcefield FF14SB [32] (for either explicit or implicit solvation) and a special-purpose AMBER forcefield FF12MC [33] (for explicit solvation only).

## 2 Theory and methods

### 2.1 Equivalence of mass scaling and time-step scaling for sampling enhancement

Scaling the total mass by a factor of *λ* for an MD simulation scales the time of the new system by a factor of $\sqrt{\lambda }$ [15], [30]. The reason is because the sole purpose to scale total mass is to improve configurational sampling. Therefore, the units of distance [*l*] and energy [*m*]([*l*]/[*t*])^2^ of low-mass simulations are kept identical to those of standard-mass simulations. This is so that the structure and energy of the low-mass simulations can be compared to those of the standard-mass simulations in order to determine relative configurational sampling efficiencies. Let superscripts ^lmt^ and ^smt^ denote the times for the low-mass and standard-mass systems, respectively. Then [*m*^lmt^]=0.1[*m*^smt^], [*l*^lmt^]=[*l*^smt^], and [*m*^lmt^]([*l*^lmt^]/[*t*^lmt^])^2^=[*m*^smt^]([*l*^smt^]/[*t*^smt^])^2^ lead to $\sqrt{10}$[*t*^lmt^]=[*t*^smt^]. It is worth noting that a conventional MD simulation program takes a time step in the standard-mass time rather than the low-mass time. Therefore, low-mass MD simulations at Δ*t*=1.00 fs^smt^ (*viz.*, $\sqrt{10}$ fs of the low-mass time) are theoretically equivalent to standard-mass MD simulations at Δ*t*=$\sqrt{10}$ fs^smt^ (*viz.*, $\sqrt{10}$ fs of the standard-mass time), if both standard-mass and low-mass simulations are carried out for the same number of time steps and if there are no precision issues in performing these simulations.

### 2.2 Molecular dynamics simulations to autonomously fold chignolin and CLN025

Chignolin or CLN025 in a fully extended backbone conformation solvated with the TIP3P water [34] with surrounding counter ions and NaCl molecules was energy-minimized for 100 cycles of steepest-descent minimization followed by 900 cycles of conjugate-gradient minimization to remove close van der Waals contacts using SANDER of AMBER 11 (University of California, San Francisco). The energy-minimized system was then heated from 0 to 277, 300 or 340 K at a rate of 10 K/ps under constant temperature and constant volume, and finally simulated in 20 unique, independent, all-atom, and classical NTP MD simulations using PMEMD of AMBER 11 with a periodic boundary condition at 277, 300, or 340 K and 1 atm employing isotropic molecule-based scaling. The fully extended backbone conformations (*viz.*, anti-parallel β-strand conformations) of chignolin and CLN025 were generated by MacPyMOL Version 1.5.0 (Schrödinger LLC, Portland, OR). The numbers of TIP3P waters and surrounding ions, initial solvation box size, ionizable residues, and computers used for the NTP MD simulations are provided in Table S1. The 20 unique seed numbers for initial velocities of Simulations 1–20 were taken from Ref. [35]. All simulations used (*i*) a dielectric constant of 1.0, (*ii*) the Berendsen coupling algorithm [36], (*iii*) the Particle Mesh Ewald method to calculate long-range electrostatic interactions [37], (*iv*) Δ*t*=1.00, 2.00, 3.16, or 3.50 fs^smt^, (*v*) the SHAKE-bond-length constraints applied to all the bonds involving hydrogen, (*vi*) a protocol to save the image closest to the middle of the “primary box” to the restart and trajectory files, (*vii*) a formatted restart file, (*viii*) the revised alkali and halide ions parameters [38], (*ix*) a cutoff of 8.0 Å for nonbonded interactions, and (*x*) default values of all other inputs of the PMEMD module. Instantaneous conformations of each simulation were saved at every 10^5^ time steps. The forcefield parameter file of FF12MC is provided in Appendix A.

### 2.3 Aggregated native state population calculation

**Fig. 1:**
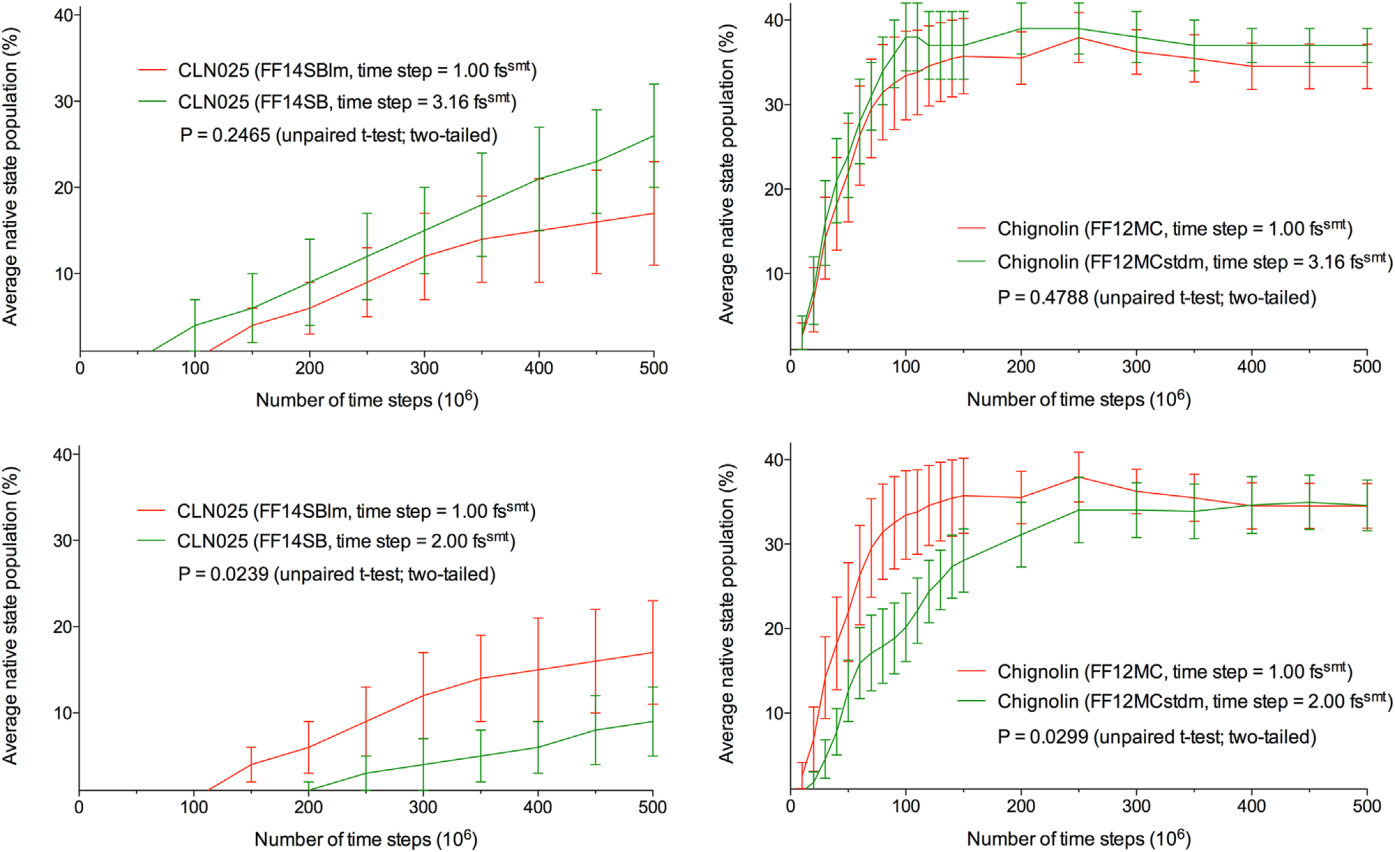
Time series of aggregated native state population for chignolin and CLN025. The two-tailed *P* values were obtained from unpaired *t* tests using the PRISM 5 program.

The native conformations of CLN025 in the NMR and crystal structures have Tyr2 and Trp9 on one side of the β-sheet and Tyr1 and Tyr10 on the other (see Ref. [1, Fig. 1A and B]). However, the reported NTP MD simulations showed that CLN025 could fold to native-like β-hairpins with Tyr1, Trp9, and Tyr10 on one side of the β-sheet and Tyr2 on the other (see Ref. [1 Fig. 1C]), or with Tyr1 and Trp9 on one side and Tyr2 and Tyr10 on the other (see Ref. [1 Fig. 1D]). Similarly, the reported NTP MD simulations also showed that chignolin could fold to the native β-hairpin with Tyr2 and Trp9 on the same side of the hairpin [31] and to native-like β-hairpins with Tyr2 on one side of the hairpin and Trp9 on the other [33].

The lowest Cα and Cβ root mean square deviation (CαβRMSD) between one of the native-like β-hairpins and the corresponding NMR structure of CLN025 is 2.08 Å, whereas the corresponding Cα root mean square deviation is 1.33 Å. The CαβRMSD between one of the native-like β-hairpins and the NMR structure of chignolin is 1.99 Å, but the corresponding Cα root mean square deviation is 1.58 Å. In this study CαβRMSDs and Cα root mean square deviations were calculated using PTRAJ of AmberTools 1.5 with root mean square fit all α and β carbon atoms to the corresponding ones in the β-hairpin NMR structure without mass weighing. To distinguish the native β-hairpins from the native-like ones, in this study conformations of chignolin and CLN025 with CαβRMSDs of ≤1.96 Å relative to their NMR structures were considered to be at the native or folded state. The time series of CαβRMSD from native conformations for chignolin and CLN025 revealed that these β-hairpins could fold into conformations with CαβRMSDs of ~1.5 Å (Fig. S1). However, the CαβRMSD cutoff for the native state was set at 1.96 Å rather than 1.50 Å because the CαβRMSD between the NMR and crystal structures of CLN025 is 1.95 Å. Otherwise, the use of a CαβRMSD cutoff of ≤1.50 Å would preclude conformations determined by crystallographic analysis that are commonly considered at the native state.

The individual native state population of chignolin or CLN025 in an MD simulation was calculated as the number of the β-hairpin conformations with CαβRMSDs of ≤1.96 Å divided by the number of all conformations saved at every 10^5^ time steps. Averaging the individual native state populations of a set of 20 unique and independent MD simulations gave rise to the aggregated native state population for the set. The standard deviation (SD) and standard error (SE) of the aggregated native state population were calculated according to Eqs. (1) and (2) of Ref. [33], respectively, wherein N is the number of all simulations, $P_{i}$ is the individual native state population of the *i*th simulation, and $\overset{¯}{P}$ is the aggregated native state population.

### 2.4 Folding time estimation using survival analysis

The folding time (*τ*_f_) or the reciprocal of the folding rate (*k*_f_) of a β-hairpin was estimated by the mean time of the β-hairpin to fold from a fully extended backbone conformation to the native conformation in 20 unique and independent NTP MD simulations, using survival analysis methods [39] from the R survival package (Therneau T.M., A Package for Survival Analysis in S, 2015, Version 2.38-3, http://CRAN.R-project.org/package=survival). The CαβRMSD cutoff of ≤1.96 Å was used to identify conformations at the native state. For each simulation with instantaneous conformations saved at every 10^5^ time steps, the first time instant at which CαβRMSD reached ≤1.96 Å was recorded as an individual folding time (Fig. S1). If a set of 20 full simulations (each performed for 500 million time steps) all captured a folding event, this set was used to calculate the mean time-to-folding using a two-step procedure. The first step used the Kaplan–Meier estimator [40], [41] with the Surv() function in the R survival package. The second used parametric survival functions—that mostly fell within the 95% confidence bounds of the Kaplan–Meier estimator—with the Surreg() function. If the mean of the Kaplan–Meier estimator was identical to the mean derived from a parametric survival function, then this survival function was used to calculate the time-course of the mean time-to-folding of the same set of simulations. If half or more than half of 20 shortened simulations (each performed for <500 million time steps) did not capture a folding event, the *τ*_f_ of these simulations was discarded because of their overly large 95% confidence interval.

## 3 Results and discussion

### 3.1 Equal sampling of simulations using low-mass at 1.00 fs^smt^ and standard-mass at 3.16 fs^smt^

It was reported previously that CLN025 did not fold from a fully extended backbone conformation to its native conformation in 10 unique, independent, all-atom, and classical 500-million–time-step NTP MD simulations at Δ*t*=1.00 fs^smt^ using FF14SB at 277 K and 1 atm [1]. When 20 such simulations were performed under the same simulation conditions, except that the forcefield was changed from FF14SB to FF14SBlm, CLN025 folded in 13 of the 20 simulations (Fig. S1A) with an aggregated native state population with SE of 17±6% (Table 1). FF14SBlm has all the parameters of FF14SB, except that atomic masses are reduced uniformly by tenfold. When the FF14SBlm simulations were repeated under the same simulation conditions, except that FF14SBlm and Δ*t* were changed to FF14SB and 3.16 fs^smt^, respectively, CLN025 folded in 16 of the 20 simulations (Fig. S1B), with an aggregated native state population including SE of 26±6% (Table 1). Plotting the aggregated native state population as a function of the number of time steps shows no significant separation between the curves of the simulations using FF14SBlm at Δ*t*=1.00 fs^smt^ and those using FF14SB at Δ*t*=3.16 fs^smt^ (Fig. 1), according to the unpaired *t*-test two-tailed *P* value of 0.2465 for the two curves. Both sets of simulations using FF14SB and FF14SBlm did not converge well, according to (*i*) the large SDs relative to the means for FF14SB and FF14SBlm listed in Table 1 and (*ii*) the result that some simulations failed to capture a folding event (Fig. S1A and B). Therefore, the *τ*_f_*s* of CLN025 were not calculated for the two sets of simulations. Nevertheless, the aggregated native state populations do show that the configurational sampling of the NTP MD simulations of CLN025 using FF14SBlm at Δ*t*=1.00 fs^smt^ is statistically equivalent to that using FF14SB at Δ*t*=3.16 fs^smt^.

**Table 1:** Folding of CLN025 in 20 NTP MD simulations using different forcefields, temperature, and time steps.

Forcefield	Temp (K)	Time step (fs^smt^)	Aggregated simulation time (μs^smt^)	Aggregated native state population (%)		
				Mean	SD	SE
FF14SBlm	277	1.00	3.16	0	1	0
FF14SBlm	277	1.00	6.32	0	1	0
FF14SBlm	277	1.00	9.48	4	10	2
FF14SBlm	277	1.00	12.64	6	15	3
FF14SBlm	277	1.00	15.80	9	19	4
FF14SBlm	277	1.00	18.96	12	22	5
FF14SBlm	277	1.00	22.12	14	24	5
FF14SBlm	277	1.00	25.28	15	26	6
FF14SBlm	277	1.00	28.44	16	27	6
FF14SBlm	277	1.00	31.60	17	28	6
FF14SB	277	3.16	3.16	0	0	0
FF14SB	277	3.16	6.32	4	11	3
FF14SB	277	3.16	9.48	6	17	4
FF14SB	277	3.16	12.64	9	21	5
FF14SB	277	3.16	15.80	12	24	5
FF14SB	277	3.16	18.96	15	24	5
FF14SB	277	3.16	22.12	18	25	6
FF14SB	277	3.16	25.28	21	26	6
FF14SB	277	3.16	28.44	23	28	6
FF14SB	277	3.16	31.60	26	29	6
FF14SB	277	2.00	2.00	0	0	0
FF14SB	277	2.00	4.00	0	0	0
FF14SB	277	2.00	6.00	0	1	0
FF14SB	277	2.00	8.00	1	4	1
FF14SB	277	2.00	10.00	3	8	2
FF14SB	277	2.00	12.00	4	11	3
FF14SB	277	2.00	14.00	5	14	3
FF14SB	277	2.00	16.00	6	15	3
FF14SB	277	2.00	18.00	8	17	4
FF14SB	277	2.00	20.00	9	19	4
FF12MCstdm	340	3.16	3.16	29	26	6
FF12MCstdm	340	3.16	6.32	36	16	4
FF12MCstdm	340	3.16	9.48	41	14	3
FF12MCstdm	340	3.16	12.64	41	13	3
FF12MCstdm	340	3.16	15.80	42	12	3
FF12MCstdm	340	3.16	18.96	42	10	2
FF12MCstdm	340	3.16	22.12	41	9	2
FF12MCstdm	340	3.16	25.28	41	9	2
FF12MCstdm	340	3.16	28.44	42	9	2
FF12MCstdm	340	3.16	31.60	41	9	2

Aggregated native state population: number of conformations from 20 simulations with CαβRMSDs of ≤1.96 Å divided by number of all conformations from the 20 simulations. All MD simulations were performed for 500 million time steps with conditions described in Section 2 and Table S1. SD: Standard deviation. SE: Standard error.

Next, 20 unique, independent, all-atom, and classical NTP MD simulations of chignolin at 300 K and 1 atm were carried out for 500×10^6^ time steps under two conditions. One used FF12MC with Δ*t*=1.00 fs^smt^, and the other used FF12MCstdm with Δ*t*=3.16 fs^smt^. FF12MCstdm has all the parameters of FF12MC, except that all atomic masses are changed to standard values. Consistent with the report that FF12MC can reduce the number of time steps of all-atom and classical NTP MD simulation required to capture of the folding of chignolin [33], in this study chignolin folded at 300 K and 1 atm from a fully extended backbone conformation to its native conformation in all 20 NTP MD simulations at Δ*t*=1.00 fs^smt^ using FF12MC (Fig. S1C) or at Δ*t*=3.16 fs^smt^ using FF12MCstdm (Fig. S1D), respectively. The aggregated native state populations with SEs of the simulations using FF12MC and FF12MCstdm are converged to 35±3% and 37±2%, respectively (Tables 2A and 2B). The convergences of the two sets of simulations are further supported by the small SDs relative to the means of the populations (Tables 2A and 2B). Using the Kaplan–Meier estimator [40], [41] (see Section 2.4), the *τ*_f_*s* of chignolin were estimated to 79 ns^smt^ (95% confidence interval of 51–123 ns^smt^) for FF12MC and 72 ns^smt^ (95% confidence interval of 47–112 ns^smt^) for FF12MCstdm (Tables 2A and 2B).

**Table 2A:** Folding of chignolin in 20 NTP MD simulations at 300 K using FF12MC.

Time step (fs^smt^)	Aggregated simulation time (μs^smt^)	Aggregated native state population (%)			Estimated folding time (ns^smt^)			
		Mean	SD	SE	Mean	LCL	UCL	Event
1.00	0.632	3	7	2	–	–	–	4
1.00	1.264	7	17	4	–	–	–	7
1.00	1.896	14	22	5	87	53	145	15
1.00	2.528	18	25	5	92	56	150	16
1.00	3.160	22	26	6	82	53	129	19
1.00	3.792	26	26	6	79	51	123	20
1.00	4.424	30	26	6	79	51	123	20
1.00	5.056	31	25	6	79	51	123	20
1.00	5.688	33	24	5	79	51	123	20
1.00	6.320	33	23	5	79	51	123	20
1.00	6.952	34	22	5	79	51	123	20
1.00	7.584	35	21	5	79	51	123	20
1.00	8.216	35	21	5	79	51	123	20
1.00	8.848	35	20	5	79	51	123	20
1.00	9.480	36	20	4	79	51	123	20
1.00	12.640	36	14	3	79	51	123	20
1.00	15.800	38	13	3	79	51	123	20
1.00	18.960	36	12	3	79	51	123	20
1.00	22.120	35	13	3	79	51	123	20
1.00	25.280	35	12	3	79	51	123	20
1.00	28.440	35	12	3	79	51	123	20
1.00	31.600	35	12	3	79	51	123	20

Aggregated native state population: number of conformations from 20 simulations with CαβRMSDs of ≤1.96 Å divided by number of all conformations from the 20 simulations. All MD simulations were performed for 500 million time steps with conditions described in Methods and Table S1. SD: Standard deviation. SE: Standard error. LCL: Lower 95% confidence limit. UCL: Upper 95% confidence limit. Event: number of simulations that captured a folding event.

**Table 2B:** Folding of chignolin in 20 NTP MD simulations at 300 K using FF12MCstdm.

Time step (fs^smt^)	Aggregated simulation time (μs^smt^)	Aggregated native state population (%)			Estimated folding time (ns^smt^)			
		Mean	SD	SE	Mean	LCL	UCL	Event
3.16	0.632	3	8	2	–	–	–	4
3.16	1.264	8	17	4	–	–	–	10
3.16	1.896	16	23	5	95	55	163	13
3.16	2.528	21	22	5	74	47	118	18
3.16	3.160	24	21	5	73	47	114	19
3.16	3.792	28	21	5	74	48	117	19
3.16	4.424	31	20	4	72	47	112	20
3.16	5.056	34	20	4	72	47	112	20
3.16	5.688	36	20	4	72	47	112	20
3.16	6.320	38	19	4	72	47	112	20
3.16	6.952	38	18	4	72	47	112	20
3.16	7.584	37	17	4	72	47	112	20
3.16	8.216	37	17	4	72	47	112	20
3.16	8.848	37	17	4	72	47	112	20
3.16	9.480	37	17	4	72	47	112	20
3.16	12.640	39	15	3	72	47	112	20
3.16	15.800	39	15	3	72	47	112	20
3.16	18.960	38	13	3	72	47	112	20
3.16	22.120	37	12	3	72	47	112	20
3.16	25.280	37	11	2	72	47	112	20
3.16	28.440	37	9	2	72	47	112	20
3.16	31.600	37	7	2	72	47	112	20

Aggregated native state population: number of conformations from 20 simulations with CαβRMSDs of ≤1.96 Å divided by number of all conformations from the 20 simulations. All MD simulations were performed for 500 million time steps with conditions described in Methods and Table S1. SD: Standard deviation. SE: Standard error. LCL: Lower 95% confidence limit. UCL: Upper 95% confidence limit. Event: number of simulations that captured a folding event.

As indicated by the unpaired *t*-test two-tailed *P* value of 0.4788 (Fig. 1), there is no significant separation between the two time series of aggregated native state population for chignolin at 300 K using FF12MC with Δ*t*=1.00 fs^smt^ and FF12MCstdm with Δ*t*=3.16 fs^smt^, respectively. The *τ*_f_ of chignolin of the FF12MC simulations is statistically equivalent to that of the FF12MCstdm simulations in terms of both mean and 95% confidence interval. These results confirm that the configurational sampling of the NTP MD simulations of chignolin using FF12MC at Δ*t*=1.00 fs^smt^ is statistically equivalent to that using FF12MCstdm at Δ*t*=3.16 fs^smt^. As to the first two aims described in Section 1, these results suggest that standard-mass NTP MD simulations at Δ*t*=3.16 fs^smt^ can actually be performed without employing the hydrogen mass repartitioning scheme. The results also suggest that, regardless of which forcefield is used, configuration sampling efficiency of standard-mass NTP MD simulations at Δ*t*=3.16 fs^smt^ is statistically equivalent to that of low-mass NTP MD simulations at Δ*t*=1.00 fs^smt^.

### 3.2 Better sampling of simulations using low-mass at 1.00 fs^smt^ than standard-mass at 2.00 fs^smt^

Repeating the above simulations of CLN025 at 277 K using FF14SB at Δ*t*=2.00 fs^smt^ showed that CLN025 folded in ten of the 20 simulations (Fig. S1E) with an aggregated native state population including SE of 9±4% (Table 1). As indicated by the unpaired *t*-test two-tailed *P* value of 0.0239 (Fig. 1), there is a significant separation between the two time series of aggregated native state population for CLN025 at 277 K using FF14SBlm and FF14SB. These series are upward and have not reached plateau (Fig. 1). In addition, the SDs are larger than the means of the FF14SBlm and FF14SB simulations. These results indicate that both sets of simulations are not well converged. Nevertheless, the unpaired *t*-test two-tailed *P* value of 0.0239 indicates that the configurational sampling of the MD simulations of CLN025 using FF14SBlm at Δ*t*=1.00 fs^smt^ is significantly better than that using FF14SB at Δ*t*=2.00 fs^smt^.

Repeating the above simulations of chignolin at 300 K using FF12MCstdm at Δ*t*=2.00 fs^smt^ revealed that chignolin folded in 19 of the 20 simulations (Fig. S1F), with an aggregated native state population including SE of 35±3% (Table 2C) and a *τ*_f_ of 147 ns^smt^ (95% confidence interval of 94–230 ns^smt^) that was estimated using the exponential survival function (see Section 2.4). As indicated by the unpaired *t*-test two-tailed *P* value of 0.0299 (Fig. 1), there is a significant separation between the two time series of aggregated native state population for chignolin at 300 K using FF12MC and FF12MCstdm. Both the mean and 95% confidence interval for the *τ*_f_ of chignolin estimated from the FF12MCstdm simulations at Δ*t*=2.00 fs^smt^ are nearly twice those obtained from the FF12MC simulations at Δ*t*=1.00 fs^smt^ (Tables 2A and 2C). These results confirm that the configurational sampling of the NTP MD simulations of chignolin using FF12MC at Δ*t*=1.00 fs^smt^ is significantly better than that using FF12MCstdm at Δ*t*=2.00 fs^smt^. As to the last aim described in Section 1, the results suggest that the low-mass NTP MD simulations at Δ*t*=1.00 fs^smt^ offer significantly better configurational sampling efficiency than the standard-mass NTP MD simulations at Δ*t*=2.00 fs^smt^.

**Table 2C:** Folding of chignolin in 20 NTP MD simulations at 300 K using FF12MCstdm.

Time step (fs^smt^)	Aggregated simulation time (μs^smt^)	Aggregated native state population (%)			Estimated folding time (ns^smt^)			
		Mean	SD	SE	Mean	LCL	UCL	Event
2.00	0.400	1	3	1	–	–	–	2
2.00	0.800	2	6	1	–	–	–	4
2.00	1.200	5	10	2	–	–	–	9
2.00	1.600	8	12	3	–	–	–	10
2.00	2.000	13	16	4	128	71	231	11
2.00	2.400	16	19	4	122	71	209	13
2.00	2.800	17	20	4	132	76	227	13
2.00	3.200	18	20	4	132	78	222	14
2.00	3.600	19	19	4	114	71	184	17
2.00	4.000	20	18	4	105	67	164	19
2.00	4.400	22	17	4	106	67	165	19
2.00	4.800	24	16	4	107	68	167	19
2.00	5.200	26	16	4	108	69	169	19
2.00	5.600	27	17	4	109	69	171	19
2.00	6.000	28	17	4	110	70	172	19
2.00	8.000	31	17	4	114	73	179	19
2.00	10.000	34	17	4	120	77	188	19
2.00	12.000	34	15	3	125	80	196	19
2.00	14.000	34	14	3	130	83	204	19
2.00	16.000	35	15	3	136	87	212	19
2.00	18.000	35	14	3	141	90	221	19
2.00	20.000	35	13	3	147	94	230	19

Aggregated native state population: number of conformations from 20 simulations with CαβRMSDs of ≤1.96 Å divided by number of all conformations from the 20 simulations. All MD simulations were performed for 500 million time steps with conditions described in Methods and Table S1. SD: Standard deviation. SE: Standard error. LCL: Lower 95% confidence limit. UCL: Upper 95% confidence limit. Event: number of simulations that captured a folding event.

### 3.3 Low-mass NTP MD simulation for configuration sampling enhancement

**Fig. 2:**
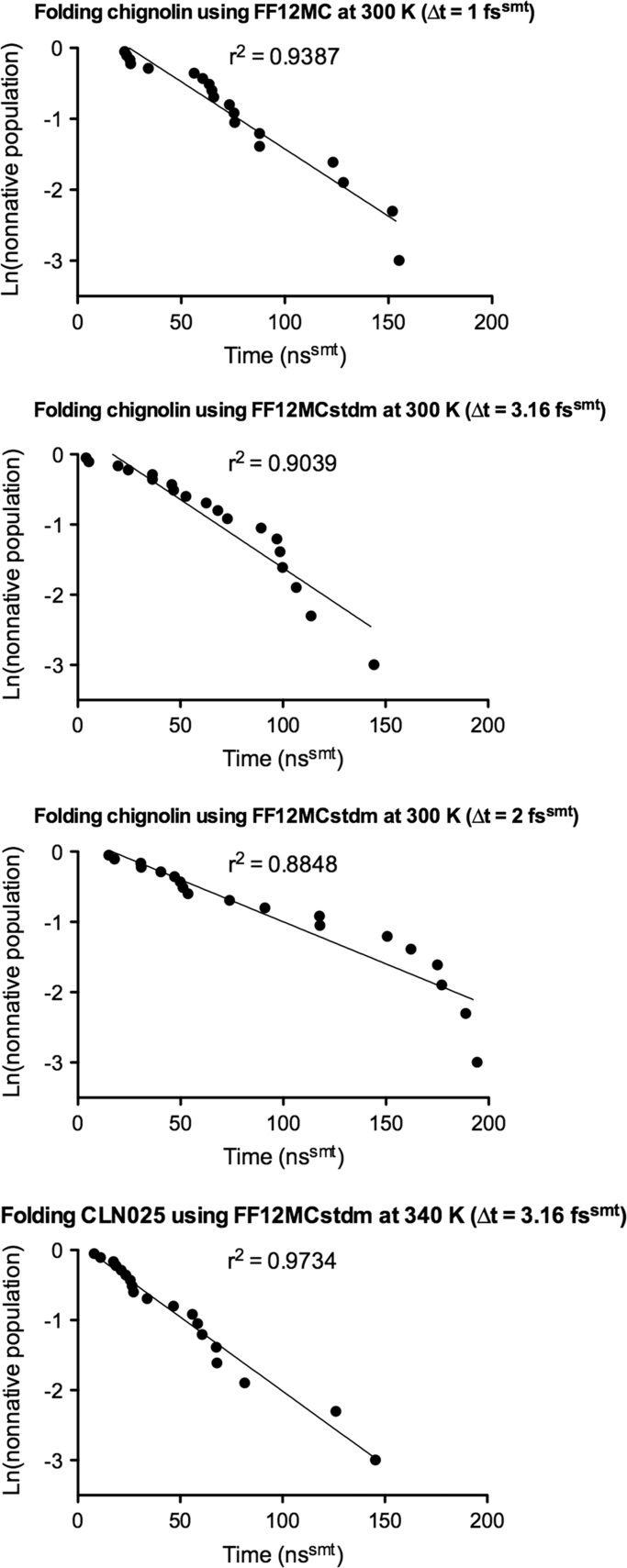
Plots of natural logarithm of the nonnative population versus simulation time. The individual folding times were taken from the data provided in Fig. S1. The linear regression analysis was performed using the PRISM 5 program.

According to the survival analysis of the folding simulations, the *τ*_f_ of chignolin estimated from the Kaplan–Meier estimator [40], [41] using the simulation data of FF12MC at Δ*t*=3.16 fs^lmt^ (*viz.,* Δ*t*=1.00 fs^smt^) was identical to the one obtained from the exponential survival function. This is evident from the linear relationship between simulation time and natural logarithm of the nonnative state population for chignolin shown in Fig. 2. The *τ*_f_ of chignolin estimated from the Kaplan–Meier estimator using the data of FF12MCstdm at Δ*t*=3.16 fs^smt^ was also the same as the one obtained from the exponential model (Fig. 2). These observations of the exponential decay of the nonnative state population over simulation time indicate that the folding of chignolin observed in the NTP MD simulations followed a simple two-state kinetics scheme of Eq. (1), wherein D and N denote the nonnative and native conformations, respectively. These results are in excellent agreement with the reported two-state folding kinetics deduced from experimental studies of chignolin, a ten-residue β-hairpin [31]. The results also agree with the generalization that a miniprotein (with residues of <100) folds according to a two-state kinetics scheme [42]. This implies that the folding rate (*k*_f_) of such a miniprotein follows the first-order rate law, namely, Eq. (2) [43]. Most importantly, the results demonstrate that the folding simulations of chignolin using Δ*t*=3.16 fs^lmt^ and Δ*t*=3.16 fs^smt^ are realistic.

(1) $$D\overset{k_{f}}{\underset{k_{u}}{⇌}}N$$

(2) $$\ln\;({\frac{\left[D\right]}{\left[D\right]}}_{0})=-k_{f}t=-\frac{1}{\tau_{f}}t$$

By contrast, the *τ*_f_ of chignolin simulated with FF12MCstdm at Δ*t*=2.00 fs^smt^ could not be estimated from the Kaplan–Meier estimator because one of the 20 simulations failed to capture a folding event (Table 2C); the relationship between simulation time and natural logarithm of the nonnative population of chignolin simulated with FF12MCstdm at Δ*t*=2.00 fs^lmt^ (*r*^2^=0.8848, Fig. 2) is not as linear as those with FF12MC at Δ*t*=3.16 fs^lmt^ and FF12MCstdm at Δ*t*=3.16 fs^smt^ (*r*^2^=0.9387 and 0.9039, respectively; Fig. 2A and B). Consequently, the *τ*_f_ of chignolin simulated with FF12MCstdm at Δ*t*=2.00 fs^smt^ has to be estimated using the exponential survival function and has a very large 95% confidence interval relative to those with Δ*t*=3.16 fs^lmt^ and Δ*t*=3.16 fs^smt^ (Table 2).

In addition, the number of time steps required to capture folding events in the simulations with Δ*t*=3.16 fs^lmt^ or Δ*t*=3.16 fs^smt^ can be reduced substantially relative to that with Δ*t*=2.00 fs^smt^. To fold chignolin at 300 K in 20 NTP MD simulations at Δ*t*=3.16 fs^lmt^, only 120×10^6^ time steps are needed for each of the 20 simulations to obtain a converged aggregated native state population. By contrast, 400×10^6^ time steps are needed to obtain the converged population for the 20 simulations of chignolin at Δ*t*=2.00 fs^smt^ using FF12MCstdm (Fig. 1). To run an NTP MD simulation of chignolin at Δ*t*=2.00 fs^smt^, 300 K, and 1 atm using FF12MCstdm and other conditions specified in Section 2.2 on a 12-core Apple Mac Pro with Intel Westmere (2.93 GHz), the average wall-clock timing is 69.52 ns^smt^ per day. The difference in number of time steps between the two sets of simulations (280×10^6^ time steps) translates to a saving of 193.33 hours of wall-clock computing time if the 20 simulations are performed in parallel or a saving of 3866.51 hours of wall-clock computing time if the 20 simulations are done in serial. To fold CLN025 at 277 K in 20 NTP MD simulations, changing Δ*t* from 2.00 fs^smt^ to 3.16 fs^lmt^ can save 210.67 or 4213.48 hours of computing time when the 20 simulations are performed in parallel or serial, respectively, according to the average timing of 56.96 ns^smt^ per day to simulate CLN025 at 277 K under other identical conditions as those for chignolin at 300 K.

There is a clear incentive from the present work to increase Δ*t* from 2.00 fs^smt^ to 3.16 fs^lmt^ or 3.16 fs^smt^ or perhaps ≥3.16 fs^smt^. However, the undesired reduction of integration accuracy can outweigh the desired reduction of computing time when Δ*t* is ≥3.16 fs^smt^ and when the hydrogen mass repartitioning scheme is not used in an MD simulation. Indeed, additional studies show that four of 20 unique and independent NTP MD simulations of chignolin at Δ*t*=3.50 fs^smt^ using FF12MCstdm at 340 K failed after 7×10^7^ time steps. But, without employing the hydrogen mass repartitioning scheme, all 20 unique and independent standard-mass NTP MD simulations of CLN025 at Δ*t*=3.16 fs^smt^ using FF12MCstdm at 340 K could be carried out successfully for 5×10^8^ time steps to capture folding events (Fig. S1G), with an aggregated native state population including SE of 41±2% and a *τ*_f_ of 55 ns^smt^ at 340 K with 95% confidence interval of 35–85 ns (Table 1 and Fig. 2). No studies have been reported to show that in practice standard-mass NTP MD simulations of regular and large proteins do not fail at Δ*t*=3.16 fs^smt^ and *T*=300–340 K. On the other hand, years of low-mass NTP MD simulations of both regular and large proteins with biological relevance [44], [45], [46], [47], [48], [49], [50], [51], [52], [53], [54], [55], [56] have already been done by this author to confirm that various proteins can be simulated successfully at Δ*t*=1.00 fs^smt^ and *T*≤340 K. Lastly, it should be pointed out that uniformly reducing atomic masses of the original system does not change the physical composition of the new system. The kinetic properties derived from low-mass NTP MD simulations can be scaled back to the properties of the original system by a factor of $\sqrt{10}$. For these reasons, low-mass NTP MD simulations at Δ*t*=1.00 fs^smt^ are presently preferred over standard-mass NTP MD simulations at Δ*t*=3.16 fs^smt^. Such low-mass NTP MD simulations can be used as a simple and generic technique to enhance configurational sampling for both kinetic and thermodynamic investigations of proteins at *T* ≤340 K.

## 4 Conclusions

The present work demonstrates that the configurational sampling efficiency of low-mass NTP MD simulations of β-hairpin folding at Δ*t*=1.00 fs^smt^ is statistically equivalent to and higher than those using standard masses at Δ*t*=3.16 and 2.00 fs^smt^, respectively. This work also shows that, without employing the hydrogen mass repartitioning scheme, standard-mass NTP MD simulations at Δ*t*=3.16 fs^smt^ and *T*=277, 300, and 340 K can be performed to capture the autonomous β-hairpin folding. While further studies may show that with sufficient computational precision standard-mass NTP MD simulations at Δ*t*=3.16 fs^smt^ might be simpler and equally effective for configurational sampling enhancement at *T* ≤340 K, the present results together with the previous results reported by this author confirm that low-mass NTP MD simulations are a simple and generic technique to enhance configurational sampling for both kinetic and thermodynamic investigations of proteins at *T* ≤340 K.
