# Supplementary material for: Low-mass molecular dynamics simulation for configurational sampling enhancement: More evidence and theoretical explanation
Source: Biochem Biophys Rep. 2015 Sep 2;4:126–33. doi: 10.1016/j.bbrep.2015.08.023 (PMC5668912; doi:10.1016/j.bbrep.2015.08.023)
Supplement: Supplementary file 1 — Supplementary material [file mmc1.pdf]

Supporting Information

Low-mass molecular dynamics simulation for configurational sampling enhancement: More evidence and theoretical explanation

Yuan-Ping Pang
Computer-Aided Molecular Design Laboratory, Mayo Clinic, Rochester, MN 55905, USA

Table S1. Numbers of TIP3P waters and ions, initial solvation box size, ionizable residues, and computers used in molecular dynamics simulations for chignolin and CLN025. ***Page 2***.

Figure S1. Smoothed time series of CαβRMSD from native conformations for chignolin and CLN025. Native state population and individual folding time are abbreviated NSP and IFT, respectively. All IFTs and time steps are converted to the standard-mass time. The smoothed curves were generated by the PRISM 5 program for Mac OS X (version 5.0d) from GraphPad Software (La Jolla, California) using 32 neighbors on each size and 6th order of the smoothing polynomial. A: Folding CLN025 using FF14SBlm at 277 K and Δt = 1.00 fs (***Page 3***). B: Folding CLN025 using FF14SB at 277 K and Δt = 3.16 fs (***Page 4***). C: Folding chignolin using FF12MC at 300 K and Δt = 1.00 fs (***Page 5***). D: Folding chignolin using FF12MCstdm at 300 K and Δt = 3.16 fs (***Page 6***). E: Folding CLN025 using FF14SB at 277 K and Δt = 2.00 fs (***Page 7***). F: Folding chignolin using FF12MCstdm at 300 K and Δt = 2.00 fs (***Page 8***). G: Folding CLN025 using FF12MCstdm at 340 K and Δt = 3.16 fs (***Page 9***).

Table S1. Numbers of TIP3P waters and ions, initial solvation box size, ionizable residues, and computers used in molecular dynamics simulations for chignolin and CLN025.

Sequence	# of H_2O	# of Na^+	# of Cl^-	Box size ($Å^3$)	Expt pH	Ionizable Residue	Computers used for the simulations
Chignolin	1281	5	3	33x34x53	5.5	ASP^3, GLU^5	Mac Pros & Xserve
CLN025	1532	6	4	35x37x55	5.7	ASP^3, GLU^5	Mac Pros & Xserve

Xserve: a cluster of Apple Xserves with 400 G5 processors (2.2/2.4 GHz). Mac Pros: a cluster of 100 12-core Apple Mac Pros with Intel Westmere (2.40/2.93 GHz).

Fig. S1A

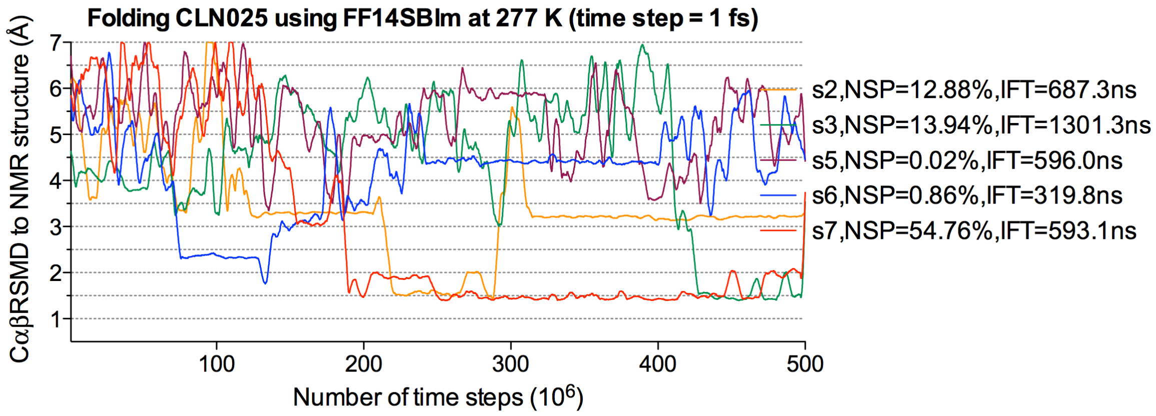

Folding CLN025 using FF14SBlm at 277 K (time step = 1 fs)
CαβRSMD to NMR structure (Å)
Number of time steps (10^6)
s2,NSP=12.88%,IFT=687.3ns
s3,NSP=13.94%,IFT=1301.3ns
s5,NSP=0.02%,IFT=596.0ns
s6,NSP=0.86%,IFT=319.8ns
s7,NSP=54.76%,IFT=593.1ns

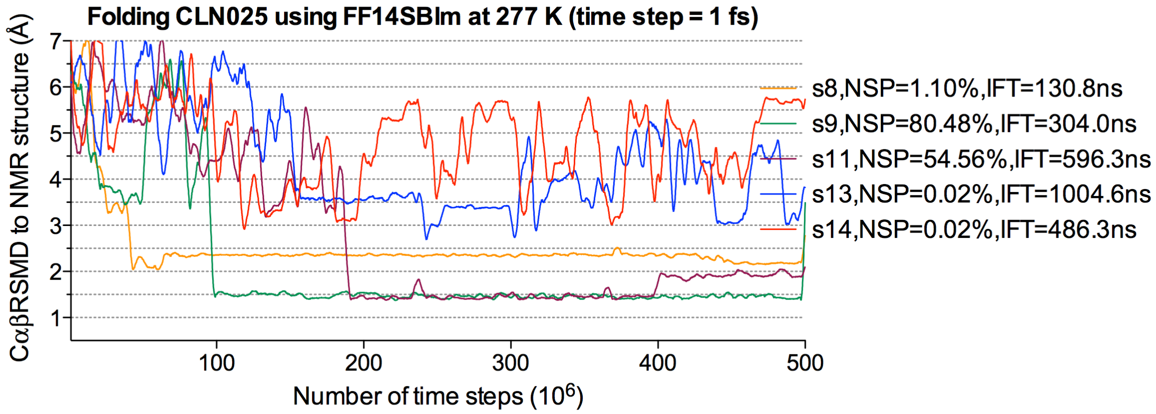

Folding CLN025 using FF14SBlm at 277 K (time step = 1 fs)
CαβRSMD to NMR structure (Å)
Number of time steps (10^6)
s8,NSP=1.10%,IFT=130.8ns
s9,NSP=80.48%,IFT=304.0ns
s11,NSP=54.56%,IFT=596.3ns
s13,NSP=0.02%,IFT=1004.6ns
s14,NSP=0.02%,IFT=486.3ns

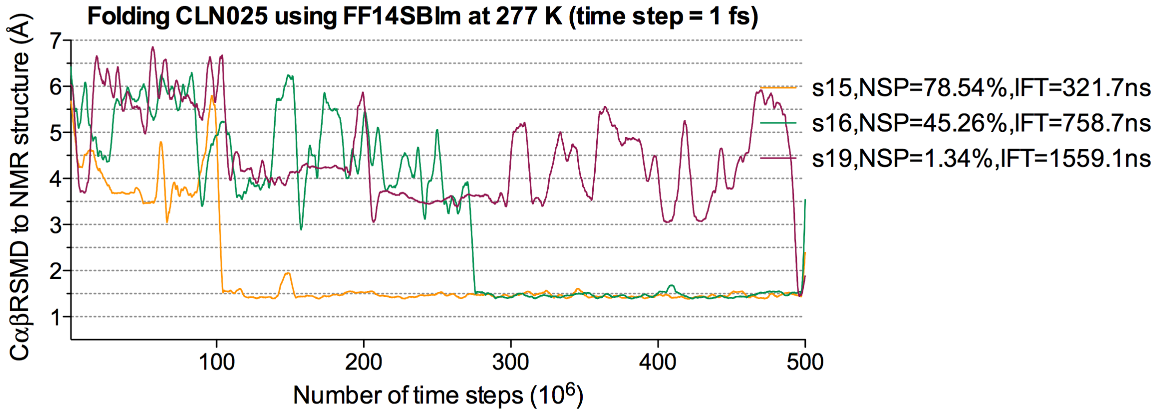

Folding CLN025 using FF14SBlm at 277 K (time step = 1 fs)
CαβRSMD to NMR structure (Å)
Number of time steps (10^6)
s15,NSP=78.54%,IFT=321.7ns
s16,NSP=45.26%,IFT=758.7ns
s19,NSP=1.34%,IFT=1559.1ns

Fig. S1B

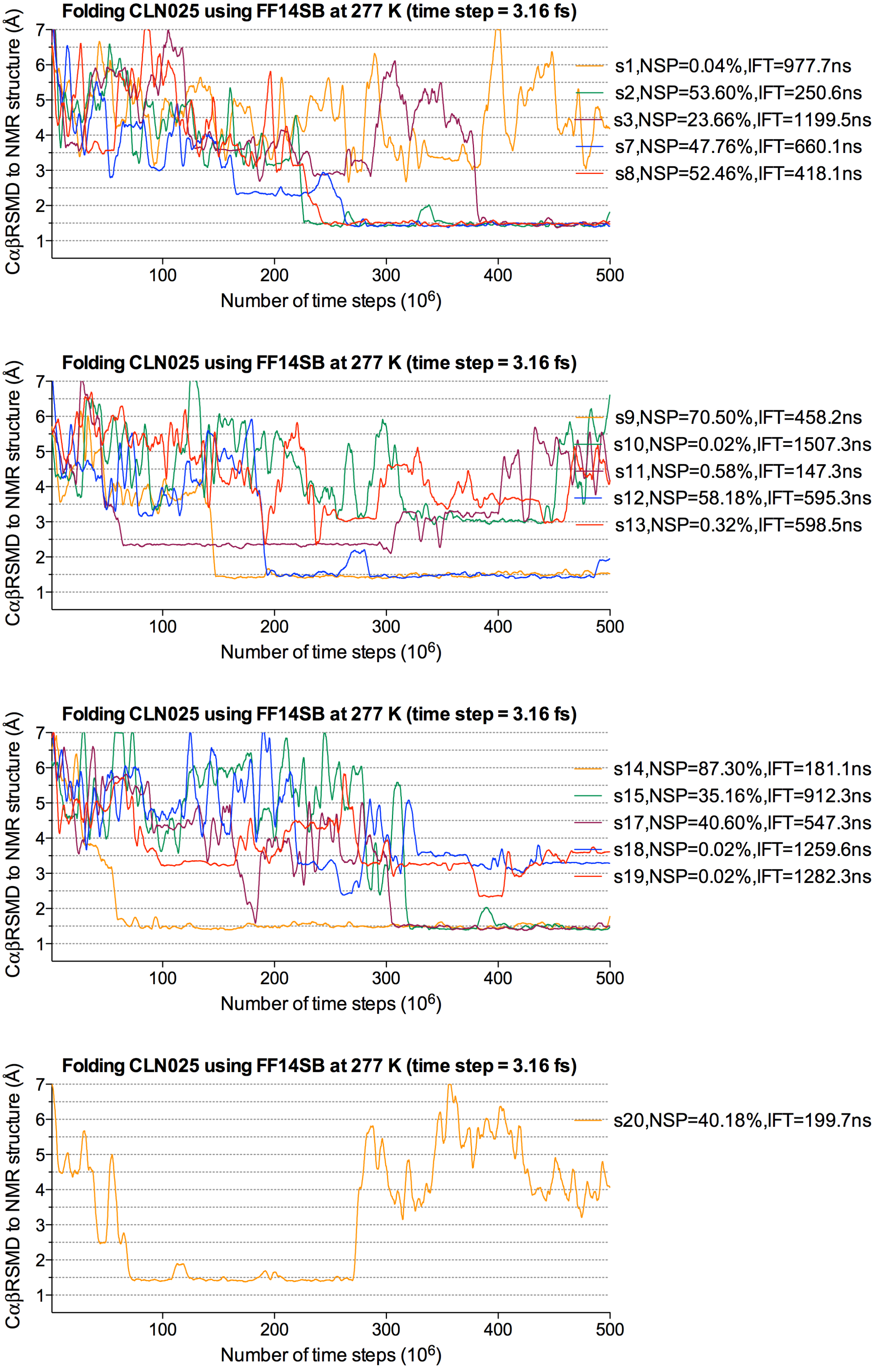
Folding CLN025 using FF14SB at 277 K (time step = 3.16 fs)
CαβRSMD to NMR structure (Å)
Number of time steps (10^6)
s1,NSP=0.04%,IFT=977.7ns
s2,NSP=53.60%,IFT=250.6ns
s3,NSP=23.66%,IFT=1199.5ns
s7,NSP=47.76%,IFT=660.1ns
s8,NSP=52.46%,IFT=418.1ns
Folding CLN025 using FF14SB at 277 K (time step = 3.16 fs)
CαβRSMD to NMR structure (Å)
Number of time steps (10^6)
s9,NSP=70.50%,IFT=458.2ns
s10,NSP=0.02%,IFT=1507.3ns
s11,NSP=0.58%,IFT=147.3ns
s12,NSP=58.18%,IFT=595.3ns
s13,NSP=0.32%,IFT=598.5ns
Folding CLN025 using FF14SB at 277 K (time step = 3.16 fs)
CαβRSMD to NMR structure (Å)
Number of time steps (10^6)
s14,NSP=87.30%,IFT=181.1ns
s15,NSP=35.16%,IFT=912.3ns
s17,NSP=40.60%,IFT=547.3ns
s18,NSP=0.02%,IFT=1259.6ns
s19,NSP=0.02%,IFT=1282.3ns
Folding CLN025 using FF14SB at 277 K (time step = 3.16 fs)
CαβRSMD to NMR structure (Å)
Number of time steps (10^6)
s20,NSP=40.18%,IFT=199.7ns

Fig. S1C

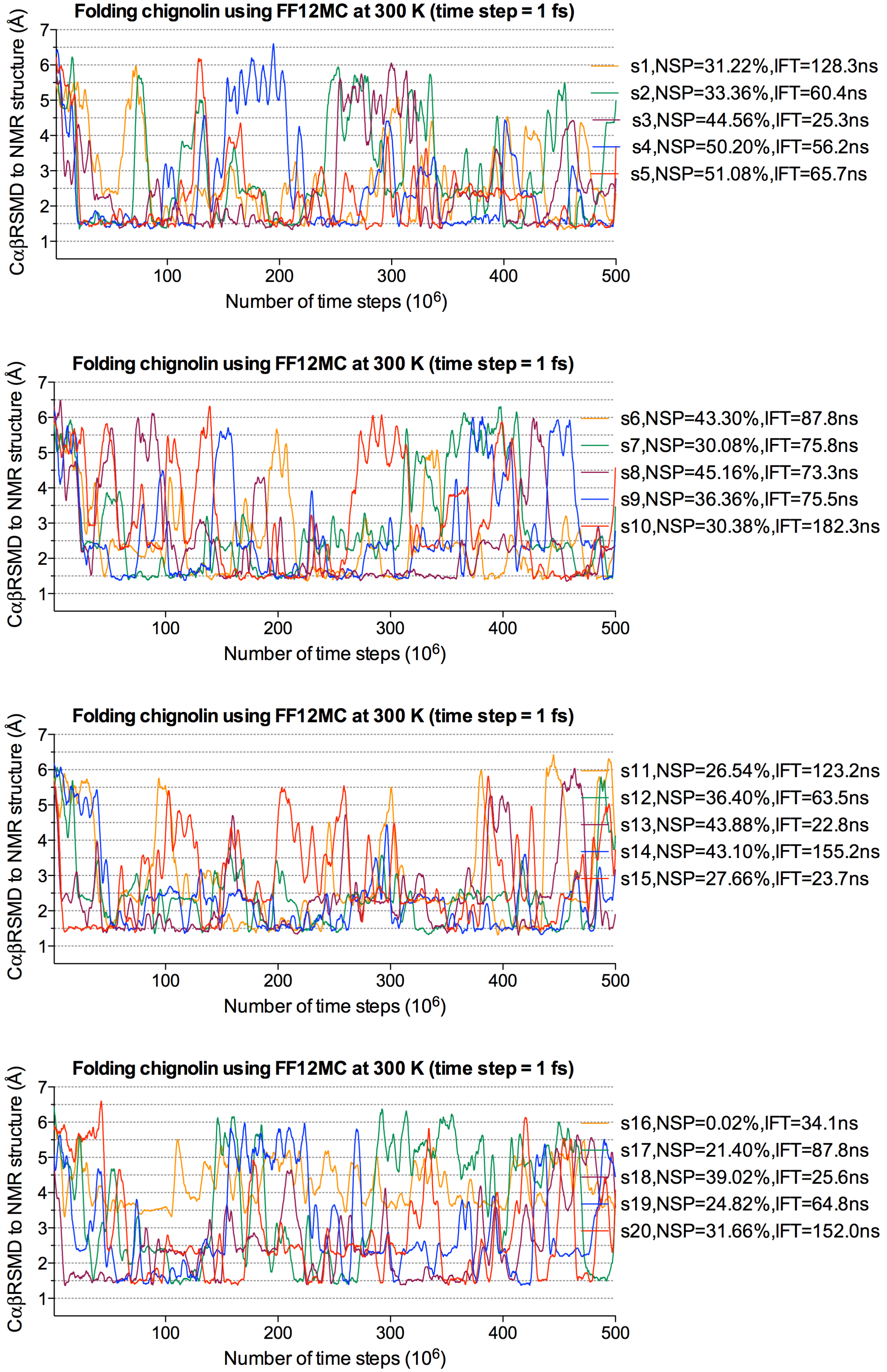

Folding chignolin using FF12MC at 300 K (time step = 1 fs)
CαβRSMD to NMR structure (Å)
Number of time steps (10^6)
s1,NSP=31.22%,IFT=128.3ns
s2,NSP=33.36%,IFT=60.4ns
s3,NSP=44.56%,IFT=25.3ns
s4,NSP=50.20%,IFT=56.2ns
s5,NSP=51.08%,IFT=65.7ns
Folding chignolin using FF12MC at 300 K (time step = 1 fs)
CαβRSMD to NMR structure (Å)
Number of time steps (10^6)
s6,NSP=43.30%,IFT=87.8ns
s7,NSP=30.08%,IFT=75.8ns
s8,NSP=45.16%,IFT=73.3ns
s9,NSP=36.36%,IFT=75.5ns
s10,NSP=30.38%,IFT=182.3ns
Folding chignolin using FF12MC at 300 K (time step = 1 fs)
CαβRSMD to NMR structure (Å)
Number of time steps (10^6)
s11,NSP=26.54%,IFT=123.2ns
s12,NSP=36.40%,IFT=63.5ns
s13,NSP=43.88%,IFT=22.8ns
s14,NSP=43.10%,IFT=155.2ns
s15,NSP=27.66%,IFT=23.7ns
Folding chignolin using FF12MC at 300 K (time step = 1 fs)
CαβRSMD to NMR structure (Å)
Number of time steps (10^6)
s16,NSP=0.02%,IFT=34.1ns
s17,NSP=21.40%,IFT=87.8ns
s18,NSP=39.02%,IFT=25.6ns
s19,NSP=24.82%,IFT=64.8ns
s20,NSP=31.66%,IFT=152.0ns

Fig. S1D

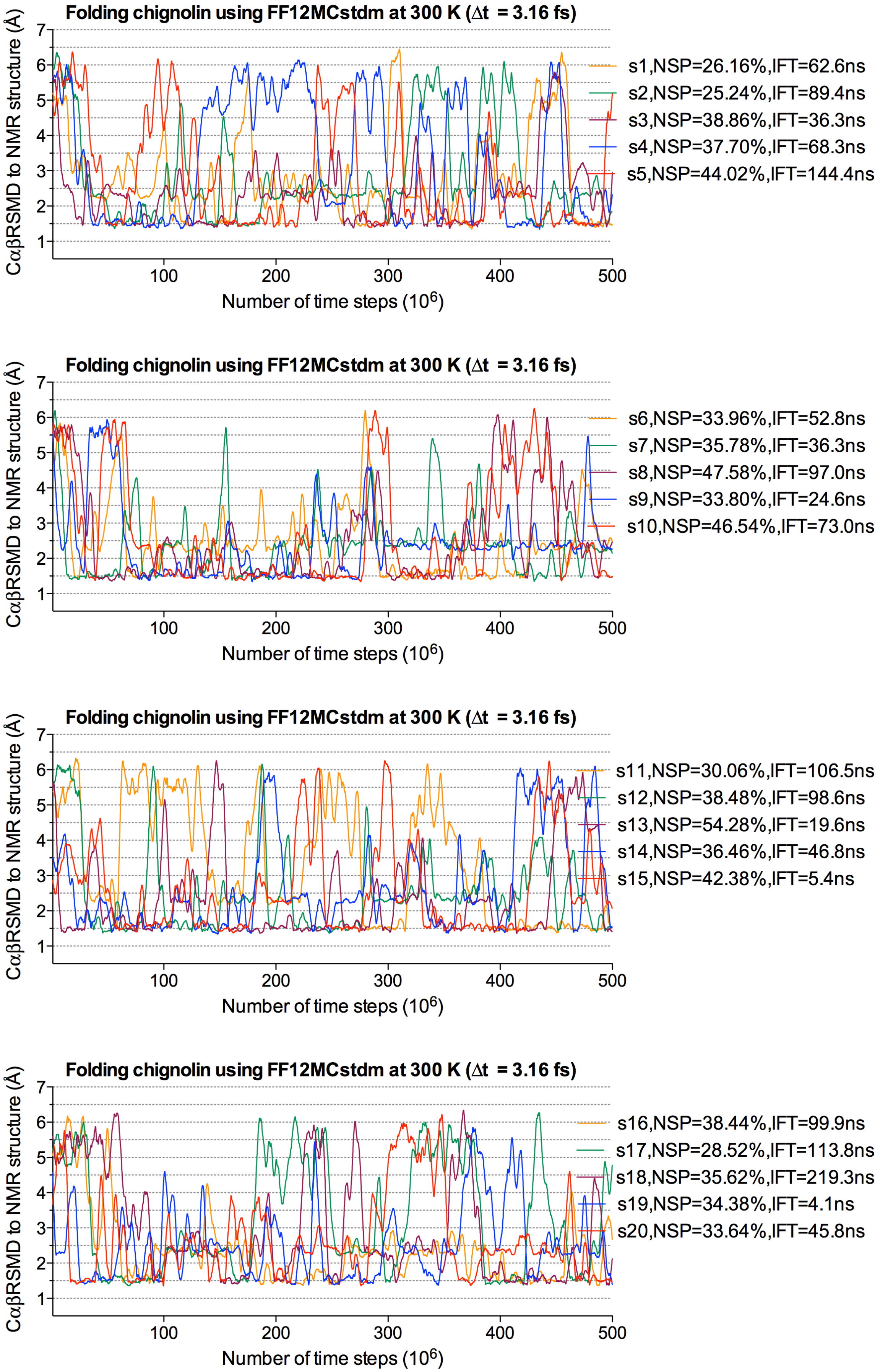

Folding chignolin using FF12MCstdm at 300 K (Δt = 3.16 fs)
CαβRSMD to NMR structure (Å)
Number of time steps (10^6)
s1,NSP=26.16%,IFT=62.6ns
s2,NSP=25.24%,IFT=89.4ns
s3,NSP=38.86%,IFT=36.3ns
s4,NSP=37.70%,IFT=68.3ns
s5,NSP=44.02%,IFT=144.4ns
Folding chignolin using FF12MCstdm at 300 K (Δt = 3.16 fs)
CαβRSMD to NMR structure (Å)
Number of time steps (10^6)
s6,NSP=33.96%,IFT=52.8ns
s7,NSP=35.78%,IFT=36.3ns
s8,NSP=47.58%,IFT=97.0ns
s9,NSP=33.80%,IFT=24.6ns
s10,NSP=46.54%,IFT=73.0ns
Folding chignolin using FF12MCstdm at 300 K (Δt = 3.16 fs)
CαβRSMD to NMR structure (Å)
Number of time steps (10^6)
s11,NSP=30.06%,IFT=106.5ns
s12,NSP=38.48%,IFT=98.6ns
s13,NSP=54.28%,IFT=19.6ns
s14,NSP=36.46%,IFT=46.8ns
s15,NSP=42.38%,IFT=5.4ns
Folding chignolin using FF12MCstdm at 300 K (Δt = 3.16 fs)
CαβRSMD to NMR structure (Å)
Number of time steps (10^6)
s16,NSP=38.44%,IFT=99.9ns
s17,NSP=28.52%,IFT=113.8ns
s18,NSP=35.62%,IFT=219.3ns
s19,NSP=34.38%,IFT=4.1ns
s20,NSP=33.64%,IFT=45.8ns

Fig. S1E

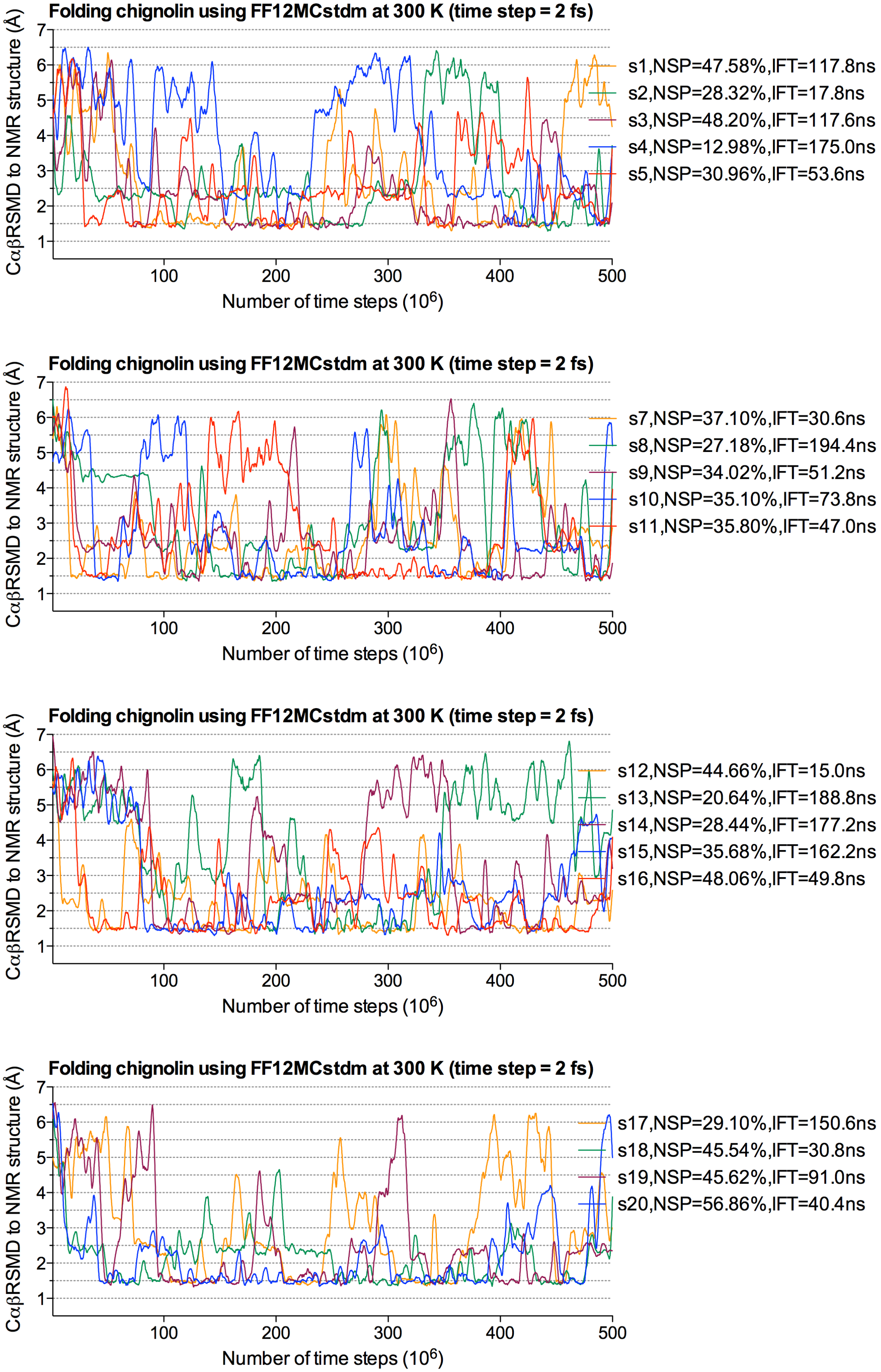
Folding chignolin using FF12MCstdm at 300 K (time step = 2 fs)
CαβRSMD to NMR structure (Å)
Number of time steps (10^6)
s1,NSP=47.58%,IFT=117.8ns
s2,NSP=28.32%,IFT=17.8ns
s3,NSP=48.20%,IFT=117.6ns
s4,NSP=12.98%,IFT=175.0ns
s5,NSP=30.96%,IFT=53.6ns
Folding chignolin using FF12MCstdm at 300 K (time step = 2 fs)
CαβRSMD to NMR structure (Å)
Number of time steps (10^6)
s7,NSP=37.10%,IFT=30.6ns
s8,NSP=27.18%,IFT=194.4ns
s9,NSP=34.02%,IFT=51.2ns
s10,NSP=35.10%,IFT=73.8ns
s11,NSP=35.80%,IFT=47.0ns
Folding chignolin using FF12MCstdm at 300 K (time step = 2 fs)
CαβRSMD to NMR structure (Å)
Number of time steps (10^6)
s12,NSP=44.66%,IFT=15.0ns
s13,NSP=20.64%,IFT=188.8ns
s14,NSP=28.44%,IFT=177.2ns
s15,NSP=35.68%,IFT=162.2ns
s16,NSP=48.06%,IFT=49.8ns
Folding chignolin using FF12MCstdm at 300 K (time step = 2 fs)
CαβRSMD to NMR structure (Å)
Number of time steps (10^6)
s17,NSP=29.10%,IFT=150.6ns
s18,NSP=45.54%,IFT=30.8ns
s19,NSP=45.62%,IFT=91.0ns
s20,NSP=56.86%,IFT=40.4ns

Fig. S1F

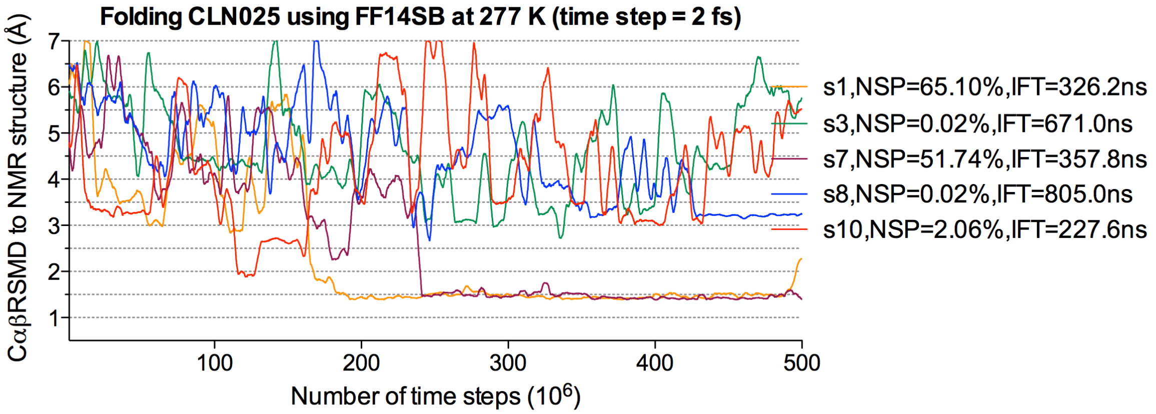
Folding CLN025 using FF14SB at 277 K (time step = 2 fs)
CαβRSMD to NMR structure (Å)
Number of time steps (10^6)
s1,NSP=65.10%,IFT=326.2ns
s3,NSP=0.02%,IFT=671.0ns
s7,NSP=51.74%,IFT=357.8ns
s8,NSP=0.02%,IFT=805.0ns
s10,NSP=2.06%,IFT=227.6ns

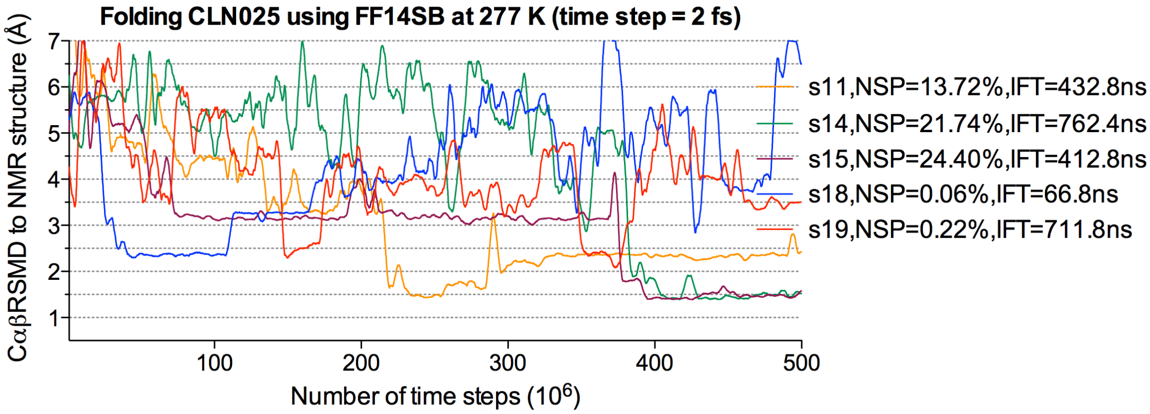
Folding CLN025 using FF14SB at 277 K (time step = 2 fs)
CαβRSMD to NMR structure (Å)
Number of time steps (10^6)
s11,NSP=13.72%,IFT=432.8ns
s14,NSP=21.74%,IFT=762.4ns
s15,NSP=24.40%,IFT=412.8ns
s18,NSP=0.06%,IFT=66.8ns
s19,NSP=0.22%,IFT=711.8ns

Fig. S1G

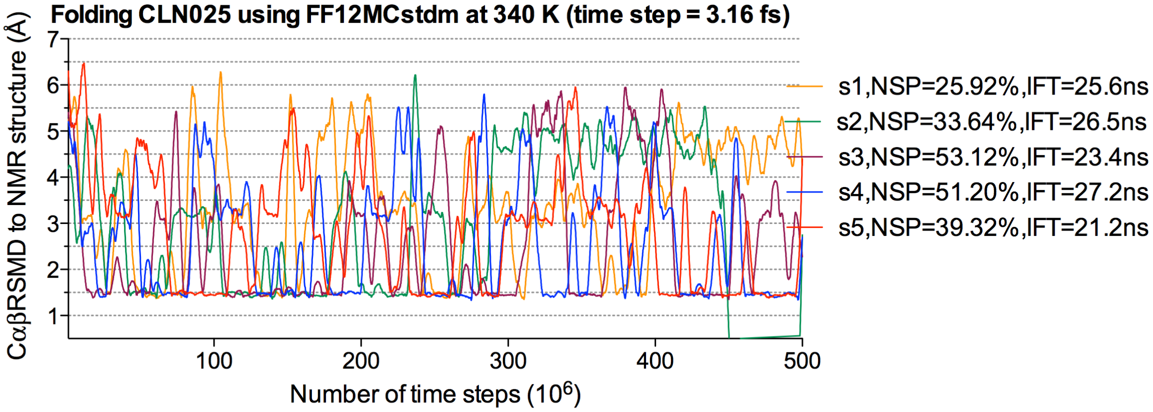
Folding CLN025 using FF12MCstdm at 340 K (time step = 3.16 fs)
CαβRSMD to NMR structure (Å)
Number of time steps (10^6)
s1,NSP=25.92%,IFT=25.6ns
s2,NSP=33.64%,IFT=26.5ns
s3,NSP=53.12%,IFT=23.4ns
s4,NSP=51.20%,IFT=27.2ns
s5,NSP=39.32%,IFT=21.2ns

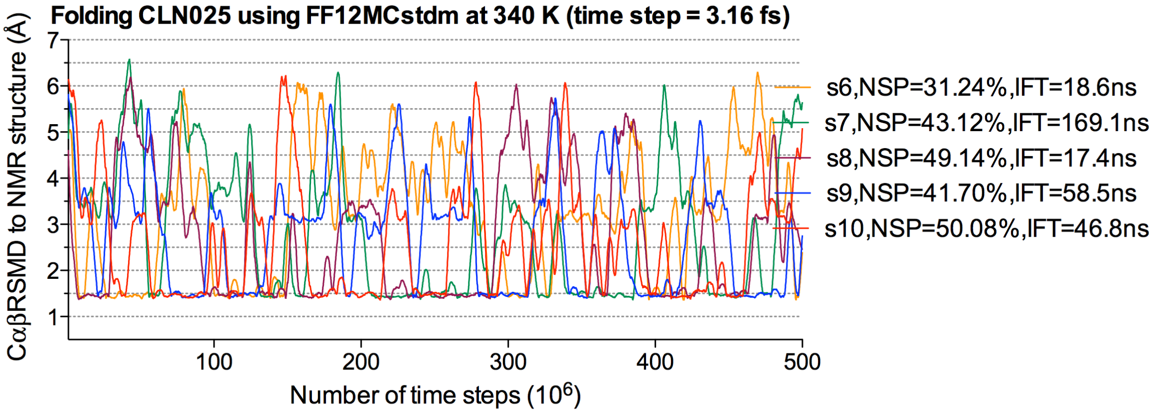
Folding CLN025 using FF12MCstdm at 340 K (time step = 3.16 fs)
CαβRSMD to NMR structure (Å)
Number of time steps (10^6)
s6,NSP=31.24%,IFT=18.6ns
s7,NSP=43.12%,IFT=169.1ns
s8,NSP=49.14%,IFT=17.4ns
s9,NSP=41.70%,IFT=58.5ns
s10,NSP=50.08%,IFT=46.8ns

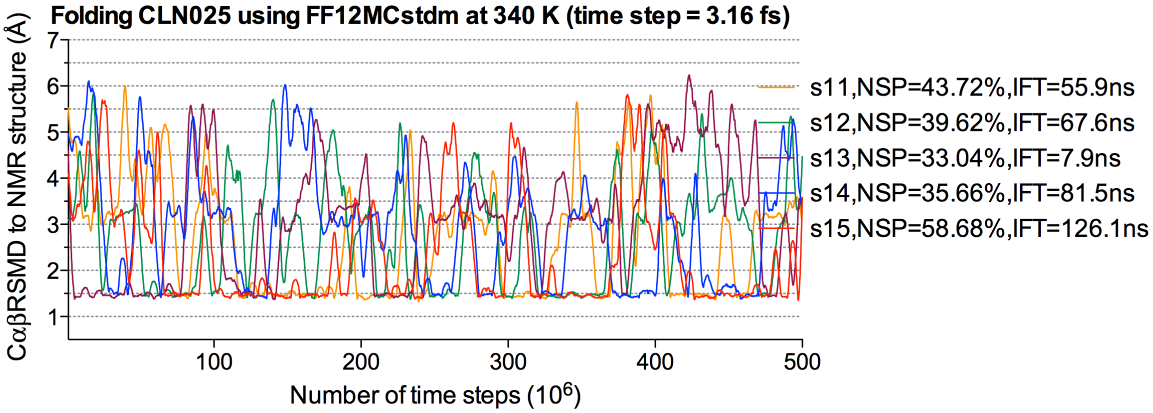
Folding CLN025 using FF12MCstdm at 340 K (time step = 3.16 fs)
CαβRSMD to NMR structure (Å)
Number of time steps (10^6)
s11,NSP=43.72%,IFT=55.9ns
s12,NSP=39.62%,IFT=67.6ns
s13,NSP=33.04%,IFT=7.9ns
s14,NSP=35.66%,IFT=81.5ns
s15,NSP=58.68%,IFT=126.1ns

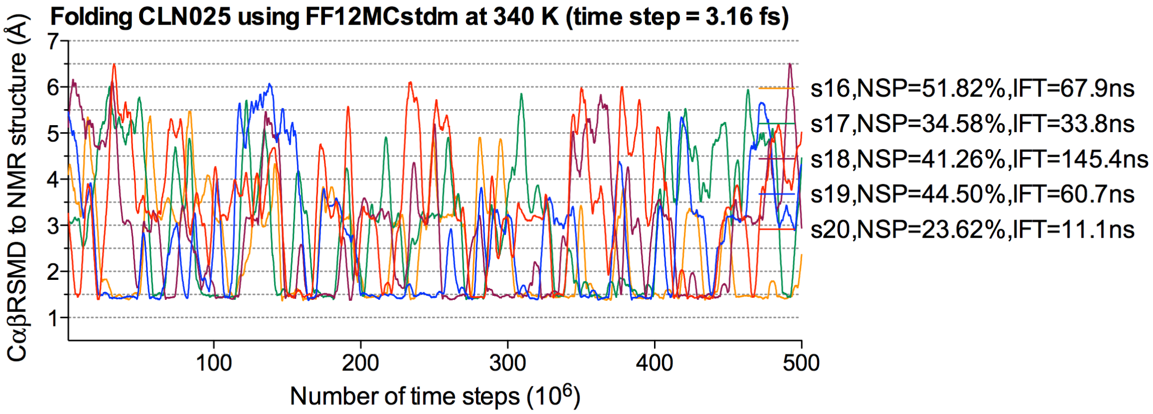
Folding CLN025 using FF12MCstdm at 340 K (time step = 3.16 fs)
CαβRSMD to NMR structure (Å)
Number of time steps (10^6)
s16,NSP=51.82%,IFT=67.9ns
s17,NSP=34.58%,IFT=33.8ns
s18,NSP=41.26%,IFT=145.4ns
s19,NSP=44.50%,IFT=60.7ns
s20,NSP=23.62%,IFT=11.1ns
